# The Differentiation of CD4^+^ T-Helper Cell Subsets in the Context of Helminth Parasite Infection

**DOI:** 10.3389/fimmu.2014.00487

**Published:** 2014-10-15

**Authors:** Tiffany Bouchery, Ryan Kyle, Franca Ronchese, Graham Le Gros

**Affiliations:** ^1^Malaghan Institute of Medical Research, Wellington, New Zealand; ^2^Victoria University of Wellington, Wellington, New Zealand

**Keywords:** CD4 T cells, helminth, differentiation, Th2, Th9, Th17, TfH

## Abstract

Helminths are credited with being the major selective force driving the evolution of the so-called “type 2” immune responses in vertebrate animals, with their size and infection strategies presenting unique challenges to the immune system. Originally, type 2 immune responses were defined by the presence and activities of the CD4^+^ T-helper 2 subset producing the canonical cytokines IL-4, IL-5, and IL-13. This picture is now being challenged by the discovery of a more complex pattern of CD4^+^ T-helper cell subsets that appear during infection, including Tregs, Th17, Tfh, and more recently, Th22, Th9, and ThGM. In addition, a clearer view of the mechanisms by which helminths and their products selectively prime the CD4^+^ T-cell subsets is emerging. In this review, we have focused on recent data concerning the selective priming, differentiation, and functional role of CD4^+^ T-helper cell subsets in the context of helminth infection. We argue for a re-evaluation of the original Th2 paradigm and discuss how the observed plasticity of the T-helper subsets may enable the parasitized host to achieve an appropriate compromise between elimination, tissue repair, containment, and pathology.

## Introduction

Helminth parasites are an extremely successful group of organisms infecting over one billion people, with some able to parasitize a host for several decades. Helminths are phylogenetically diverse, with a broad range of migration patterns and life cycles, and are spread across three phyla: nematodes, trematodes, and cestodes. Despite their diversity, the mammalian immune response against these helminths is consistently of the type 2 phenotype characterized by IgE antibody production, eosinophilia, mastocytosis, and specific forms of fibrotic wound repair under the control of the cytokines interleukin-4 (IL-4), IL-5, and IL-13. More recently, the ongoing refinement of our understanding of the type 2 immune response and the recent description of new T-helper cell subsets, force us to re-evaluate the guiding paradigm that would be informative to future studies of type 2 responses in the context of helminth infection.

## Role of Type 2 Immunity in Helminth Infection

The role of type 2 immune responses in immunity against helminths was initially revealed in studies that observed an inverse correlation between levels of parasitemia and the expression of the Th2 cell-derived cytokine IL-4 against the nematode *Trichinella spiralis* (1, 2). These ideas have been further developed in experimental models that show that signaling through IL-4Rα or the IL-4 signaling pathway STAT6 can play important roles in expulsion of, or protection against, the nematodes *Heligmosomoides polygyrus* (3)*, Nippostrongylus brasiliensis* (4, 5)*, Trichuris muris* (6), the trematode *Schistosoma mansoni* (7), and the cestode *Mesocestoides corti* (8). Although the helminth infection-induced immune effector response normally associated with IL-4 is the production of IgE antibody, the *Trichinella spiralis* experimental infection model is the only one to show a requirement for IgE in protection (9). It should also be noted that IL-4 mediated responses may not always be protective as seen in the study showing STAT6^−/−^ mice have greater resistance to the cestode *Tenia crassiceps* (10).

IL-4 is not the only Th2 derived cytokine that can signal through STAT6. The type 2 cytokine IL-13 has been shown to play a key protective role in many helminth infections, particularly in the expulsion of parasites from the gut by mediating goblet cell mucous production and smooth muscle cell contraction sometimes referred to as the “weep and sweep effect” (11). Macrophages express IL-4Rα, and signaling via both IL-4 and IL-13 can induce an alternately activated phenotype. Alternately activated macrophages produce factors that contribute to the repair of tissues damaged by infection (12); they have also been shown to be required for protective responses against some nematode infections (13).

IL-5 is the third cytokine commonly associated with type 2 immune responses and the Th2 cell subset specifically. The main function of this cytokine is the expansion of eosinophils from the bone marrow (14) with overexpression of IL-5 leading to decreased larvae numbers in primary infections of the nematodes *N. brasiliensis* and *Angiostrongylus cantonensis* (15). Genetic deletion or antibody neutralization of IL-5 or the IL-5 receptor α (IL-5Rα) show a requirement for IL-5 and eosinophils in protective immunity against secondary infections of *Strongyloides stercoralis*, *Strongyloides venezuelensis*, and *Onchocerca lienalis* (16). Eosinophils and IL-5 have also been shown to play an important role in vaccine-induced protection against *Litosomoides sigmodontis* (17).

Recent work has reported that the Th2 cell population is heterogeneous, containing some subpopulations of Th2 cells that produce both IL-5 and IL-13 in the absence of concomitant IL-4 expression (18) and also some subpopulations of Th2 cells that are IL-5^+^ or IL5^−^, while expressing IL-4 (19). Furthermore, a study by Liang et al. demonstrated that production of IL-4 and IL-13 is spatially separated with IL-13 being poorly expressed at low levels by lymph node (LN) CD4^+^ T-cells but strongly expressed by CD4^+^ T-cells found in the lung (20). These data, along with others, showing that LN CD4^+^ T-cells expressing IL-4 in response to *H. polygyrus* are primarily of the Tfh phenotype (T follicular helper) (21), negates the view that IL-4 production is a sufficiently comprehensive marker for all the T-helper cell subsets activated during the full expression of a type 2 immune response. It also raises the issue of how we need to have a broader view of how CD4^+^ T-helper cells should be defined and identified as contributors to type 2 immunity.

## The Priming of Type 2 T-Cells in Helminth Infection

**Figure 1:**
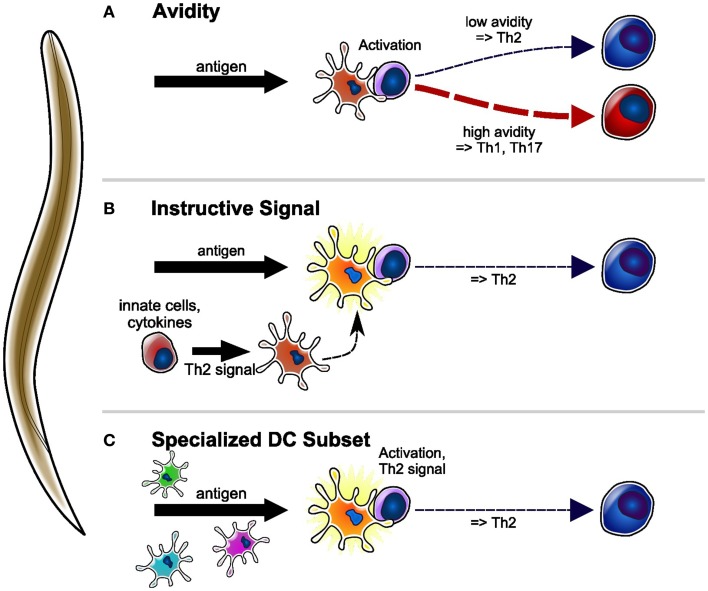
**Proposed models of Th2 differentiation induced by helminth parasite antigens presented by DC**. **(A)** Antigen taken up by DC is presented to specific CD4 + T-cells. Low-avidity interactions between CD4 + T-cell and DC result in the priming of Th2 cells, whereas high avidity interactions result in the priming of Th1 or Th17 cells. **(B)** DC conditioned by parasite-induced innate cytokines/cells, or directly conditioned by parasite products, acquire the ability to prime Th2 responses. Th1 and/or Th17 responses are initiated by DC conditioned by other innate signals. **(C)** A specific DC subset that is uniquely able to take up parasite material is programed to prime Th2 immune responses. The priming of other T-cell phenotypes requires other DC subsets.

The cellular and molecular mechanisms that lead to the priming of type 2 T-cells during helminth infections are not well understood. The IL-4 producing Th2 cell has received the most attention in this regard, with early expression of *Il4* used as a marker of pre-commitment to a T-helper cell of the type 2 lineage. Although IL-4 has been clearly demonstrated to promote overwhelming polarization and differentiation of naïve CD4^+^ T-cells into Th2 *in vitro* (22), it has been difficult to identify the *in vivo* sources of IL-4 that are able to affect the initial Th2 cell priming. More importantly, *in vivo* studies indicate that Th2 cells can be effectively primed even in the absence of IL-4- and STAT6-dependent signaling (23–25), thus suggesting that signals other than IL-4 must be operating physiologically. The difficulty in identifying such signals has led to the formulation of a number of models of Th2 priming, which are briefly outlined in Figure 1 below, and linked to available evidence in helminth infection models. Some of this evidence has recently been reviewed (26) and thus is only briefly discussed here.

Several lines of experimental evidence suggest that low-avidity interactions between T-cells and antigen presenting cells (APC) favor the development of Th2 immune responses (27) (Figure 1A). Recent work in support of this possibility includes studies of differential activation of T-cells using antigens of varying affinities or concentrations (28, 29), different T-cell–APC ratios (30) and, more recently, the reduced APC-naïve T-cell contact time observed when T-cells are primed *in vivo* in conditions that favor type 2 rather than type 1 differentiation (31). This model of Th2 priming is strongly supported by many elegant studies in well-defined experimental systems, often using homogeneous T-cell populations expressing clonal T-cell receptor (TCR). However, models in which T-cell avidity is the sole driver of T-helper cell differentiation are difficult to reconcile with the observed prevalence of specific phenotypes of immune response during infections, when complex mixtures of antigens interact with T-cells of a range of specificities such as those found in polyclonal repertoires (32). The wide range of T-cell avidities involved in such situations would predict that the resulting immune response should include a mixture of T-cell phenotypes, but this is not normally observed. It is possible that APC-derived signals, which are differentially invoked by infectious agents such as intracellular versus helminth parasites, might be involved in modulating the strength of CD4^+^ T-cell activation, thereby resulting in preferential differentiation of a certain T-helper subset (33–36). At this stage, the precise contribution of the avidity of APC-T-cell interaction to the induction of Th2 immune responses to helminth parasites remains to be determined.

An alternative possibility is that Th2 cells might arise as a “default,” in situations where the cytokines that normally direct T-cells to differentiate into other phenotypes, such as Th1 or Th17, are absent. Indeed, although parasites still require co-stimulation in order to induce optimal Th2 priming (37, 38), they mostly lack the microbes-associated molecular patterns (MAMPs) that are key to strong APC activation, co-stimulatory molecule expression, and IL-12 secretion (39–41). Alternatively, low-avidity APC-T-cell interactions may fail to sustain the CD40L expression necessary for optimal IL-12 production, as is observed with antigen-MHCII ligands that engage the TCR with low avidity. While this scenario could be consistent with the low-avidity model discussed above, it is not consistent with experiments in IL-12 KO mice showing that lack of this cytokine does not result in activation of Th2 responses (42), or with many experiments showing that helminth products suppress IL-12 production (43, 44) and instruct DC to initiate Th2 immune responses (39, 45–49). Thus, the overall evidence in support of this model remains limited.

Third, Th2 immune responses may be initiated by instructive signals from the APC. These signals may be acquired by APC through interactions with innate cell populations or mediators, or may be constitutively expressed by specialized subsets of APC. The nature of the APC that prime Th2 responses has been debated, with mast cells (50), B-cells (51), and basophils (52) being suggested as necessary, or even sufficient (53, 54), for *in vivo* Th2 induction in various models of immune responses including helminth infections. However, later experiments in a *S. mansoni* model provided clear evidence that DC depletion was sufficient to ablate Th2 induction, while basophil depletion had no effect (55). While it may be reasonable to hypothesize that parasites with different life cycles and target tissues may also involve different APC populations, a key role of DC in priming Th2 responses is consistent with a wealth of experiments in other types of T-cell responses, and also with older experiments in B-cell-deficient mice (56, 57). Altogether, the weight of experimental evidence appears to support DC as being the primary, and possibly the sole, initiator of Th2 immune responses. This conclusion then leads to the question: if DC is the principal APC population that primes Th2 responses, which properties enable them to do so, and how are these properties acquired?

The interaction of DC with helminth products has been extensively examined using DC generated in culture or, less frequently, *ex vivo* spleen DC. These experiments were highly informative and revealed, among other things, the limited upregulation of co-stimulatory molecules on DC by helminth products (36, 40), the role of CD40 (58, 59), OX40L (60), IL-4, and IL-12 (58), and the effects of treatment with TLR ligands (61). However, these results are limited by the fact that the cultured DC used in many of those experiments may not have a physiological equivalent *in vivo*, and splenic DC are not necessarily involved in the immune response to the helminths of interest. These results must, therefore, be extended and confirmed using *in vivo* models, which involve relevant antigens, DC subsets, and routes of exposure.

As discussed above, helminths and their products can directly or indirectly condition DC to prime Th2 responses (Figure 1B). On the basis of current evidence, the best candidate molecule associated with DC’s ability to program responding CD4^+^ T-cells to a Th2 phenotype is OX40L (62, 63). Blocking OX40L on human DC also suppresses their ability to prime IL-4-, IL-5-, and IL-13-producing T-cells *in vitro* (64); however, studies using transfer of mouse DC conditioned *in vitro* using SEA (*S. mansoni* Soluble Egg Antigen) suggest that OX40L may also control T-cell expansion *in vivo* (60). Expression of OX40L on DC can be induced by the innate cytokines TSLP (Thymic Stromal Lymphopoietin), IL-25, and IL-33 (64–66), which can be released by damaged epithelia. In addition, some parasite products (39, 67) and CD40 signaling can also cause upregulation of OX40L expression (60, 67). Accordingly, innate cytokines were found to be dispensable for IL-4 production after infection with several – but not all – helminths, suggesting that parasite products may be able to bypass the requirement for innate cytokines (49, 68, 69). Perhaps more surprisingly, it was also found that OX40^−/−^ mice can generate IL-4 responses to *N. brasiliensis* (70), whereas IL-4 responses to *H. polygyru*s are reduced but not ablated, suggesting a variable ability of helminth parasites to bypass or replace the requirement for OX40L co-stimulation. Our experiments comparing Th2 immune responses induced by various agents suggest that helminths may not be unique in their ability to bypass the TSLP/OX40L axis, and that HDM given intradermally can also induce TSLP-independent Th2 responses (48, 71). These results may suggest that route of exposure has a substantial effect on the involvement of innate cytokines in the resulting Th2 immune response. While this possibility is plausible, it must be reconciled with information on the innate environment in different tissues and, most importantly, must be addressed and confirmed experimentally.

Finally, Th2 responses may require a specialized DC subset that is specifically programed to carry out this function (Figure 1C). The concept of a Th2-dedicated DC subset is not new (72), and may fit with the DC heterogeneity that is gradually being revealed in all tissues. In line with this notion, recent publications identified a subset of skin DC, expressing the carbohydrate-binding molecule CD301b (73) together with PD-L2 (Programed cell death ligand 2), which is preferentially able to take up “Th2-inducing” antigens. These CD301b^+^ DC required IRF4 (interferon regulatory factor 4) for their development, and were necessary for the priming of IL-4- and IL-13-producing T-cells *in vivo* and *in vitro* (74–76), but not for IL-4-producing Tfh. Interestingly, while essential, purified CD301b^+^ DC were unable to prime Th2 responses *in vitro* or upon transfer into recipient mice, suggesting that another cell population was also required (75). Similar to those reports, our studies examining the DC populations involved in the immune response to non-viable *N. brasiliensis* larvae given subcutaneously (48) found that parasite material was preferentially taken up by a migratory population of CD11b^+^ DC also expressing CD301b, PD-L2, and IRF4. However, unlike the studies above, we were able to show that transfer of total migratory DC from mice exposed to non-viable *N. brasiliensis* larvae could prime Th2 responses in naïve mice, and that this property was independent of the antigen specificity of the responding T-cells. Importantly, we also found that DC from untreated mice could induce T-cell expansion *in vivo*, but not Th2 responses. Thus, our results suggest that exposure to helminths and the attending innate signals are important factors in conditioning DC for Th2 priming. As our experiments used transfer of mixed populations of DC, we cannot conclude on whether Th2 priming was the property of one specific DC population, or whether it required the cooperation of several DC subsets. In any case, the powerful Th2-inducing properties of helminth parasites are likely to provide a useful model in which to investigate functional DC subsets in airway and intestinal tract, and their relationship to CD301b^+^ DC. The report that IRF4 expression by DC is necessary for the priming of Th2 responses to inhaled allergens (77) suggests that DC populations able to prime Th2 responses in different tissues may share some common features. Whether these observations also apply to the other CD4^+^ T-helper cell subsets has yet to be determined.

## New T-Helper Cell Subtypes Associated with Helminth Infection

The development of new technologies, including multicolor flow cytometry and the engineering of fate-mapping and cytokine reporter mice, has lead to the discovery and definition of new subsets of T-helper cells in the past 10 years, namely Th17, Th22, Th9, Tfh and the recently suggested ThGM (T-helper producing GM-CSF). The roles of these newly described subsets, especially in the context of helminth infection, have not been fully elucidated. Here, we review the findings to date in this area, and outline the future questions that will be important to address. The role of Tregs in helminthes infection has been extensively reviewed (78–80) recently and so will not be discussed in this review.

## Th9 Cell Subset in Helminth Infection

IL-9 was originally associated with the Th2 immune response, with reports that IL-9 expression by CD4^+^ T-cells was high in Th2-pre-disposed, susceptible BALB/c mice infected with *Leishmania major*, and lower in resistant C57BL/6 (81). This view was further confirmed in anti-helminth immunity a few years later (21, 82, 83). However, recent work has shown that IL-9 and IL-4 are rarely produced by the same T-cells, thus suggesting that IL-9-expressing cells represent a discrete T-helper subset, termed Th9 (84, 85). However, both Th17 and iTregs cells have also been shown to be able to produce detectable amounts of IL-9, though not to the extent of Th9 cells. The status of Th9 as a T-helper subset has been further strengthened by the discovery that IL-4 and TGF-β were permissive for Th9 subset differentiation (84) with PU.1 defined as the necessary transcription factor. It is important to note that even if Th9 is now considered as a distinct subset, its proximity to cells of the Th2 subset is re-enforced by both the demonstration that IL-4 is needed to differentiate Th0 cells into Th9, and by the observation of inter-conversion of Th2 into Th9 in presence of TGF-β (84). More recently, however, IL-1 family members have been shown to be able to trigger an IL-4-independent Th9 differentiation (86).

IL-9’s role in helminth infection has recently been suggested in two consecutive studies showing that IL-9 transgenic mice infected with either *T. muris* or *T. spiralis* had an increased Th2 response and faster expulsion of the parasite from the intestine (82, 83). In these studies, increased mast cell and eosinophil numbers correlated with increased IL-9 levels, and were suggested as downstream cellular effectors. However, further studies showed that mice vaccinated with IL-9-OVA complex recruited similar numbers of mast cells and eosinophils to the gut of *T. muris*-infected mice, even though the treatment inhibited expulsion of the parasite (87). No other change to the type 2 response was noted. Conversely, vaccination with IL-9-OVA complex did facilitate expulsion of *T. spiralis*, illustrating that despite the general association of type 2 immunity with helminths, the effectiveness of each subtype is fine-tuned to the parasitic species involved (88). In this regard IL-9 has been shown to increase jejunal muscle contractility, and in IL-9-OVA complex vaccinated mice infected with *T. muris* contractility of the intestine was significantly decreased (88).

More recently, IL-9 has been shown to be produced by T-cells during *N. brasiliensis* infection (89, 90) with adoptive transfer of Th9 cells shown to be sufficient for mediating worm expulsion (89). However, the modest differences in worm burden detected between infected IL-9^−/−^ and wild type mice, the high experimental variability, and the need for timing differences for expulsion to be considered indicate that further work is needed to determine the role of IL-9 in the context of immunity to reinfection (90).

In a *Trichuris* model, using CD4dnTGFbRII mice (which lack Th9 cells) evidence suggested that Th9 cells are required for efficient expulsion of the parasite (84). The susceptible phenotype was associated with a decrease in mastocytosis and IL-9 expression in the mesenteric LN. However, the presence and frequencies of IL-9 producing CD4^+^ T-cells *in vivo* was not assessed in this study and the CD4dnTGFbRII mice had a decreased IL-4 response but normal IL-13 response to *Trichuris*, indicating possible defects other than the lack of IL-9, that could contribute to susceptibility. Finally, IL-9 has a role in controlling fibrosis through upregulation of prostaglandin E2 (PGE2), a well-known anti-fibrotic molecule (91), and has been recently shown to be essential to mucosal wound healing in an oxazolone-induced colitis model, through the upregulation of claudin-2 in intestinal epithelial cells (92).

## Th17 Cell in Helminth Infection

Th17 was identified as a subset distinct from Th1 and Th2 differentiation in 2005 (93), based on cellular production of IL-17 in the absence of IFN-γ or IL-4. These cells are considered proinflammatory as they express high levels of their signature cytokine IL-17, as well as IL-22, IL-6, and TGF-β, all under the control of the master transcription factor RORγt. Combinations of IL-23, TGF-β, IL-6, and IL-21 direct the differentiation of Th17 cells from naïve CD4^+^ T-cells (94). Th17 cells exacerbate experimental autoimmune encephalomyelitis (EAE) (95) but also contribute to protection against models of fungal infection (96).

The role of Th17 in helminth infection has principally been studied in *S. mansoni* models, where it has been strongly associated with infection-induced immunopathology. The pathologic role of IL-17 in helminth infection was originally recognized by its association with the development of hepatointestinal perioval granulomas caused by *S. mansoni* infection. In these early studies, CD4^+^ T-cells were known to be required for the development of the pathology (97, 98), and under the Th1:Th2 paradigm, the role of IL-17 was interpreted as being part of the Th1 immune response causing increased pathology versus a less destructive Th2-dominant response (99).

Further, in an interesting study of mouse strain related susceptibility to pathology (100), it was found that pathology was diminished in IL-12p40^−/−^ mice but not IL-12p35^−/−^ and that IL-17 but not IFN-γ levels correlated with disease, indicated that pathology was likely controlled by Th17 cells in an IL-23 dependent manner (100). Further evidence for this was provided by genetic depletion of IL-23 and disruption of IL-1β signaling leading to decreased IL-17 levels and decreased pathology (101). Furthermore, CD4^+^ T-cells from TCR transgenic mice recognizing *Schistosoma* antigen Sm-p40 expressed IL-17 when stimulated by DCs loaded with *Schistosome* eggs (102). Antibody neutralization of TGF-β lead to decreased plasma levels of IL-17 and a reduced worm burden, although this may have also changed other parameters including Treg populations (103). IL-17 from RORγt expressing Th17 cells was also associated with the severe pathology seen in natural infection with *Schistosoma japonicum* with antibody neutralization of IL-17 leading to diminished neutrophil infiltration in the liver and reduced hepatic and pulmonary pathologies (104–106). IL-17-associated pathology is also evident in human studies with children infected with *Schistosoma haematobium* having a higher circulating Th17:Treg cell ratio than those children infected but pathology-free, mirroring the ratios seen in high-pathology CBA mice compared to mild pathology C57Bl/6 (107). While studies identifying T-cells producing IL-17 in *Schistosoma* infected tissues show that most are CD4^+^ T-cells, the link to the expression of the transcription factor RORγt has been rarely attempted, also the downstream mechanisms of IL-17-associated immunopathology remain largely unknown, with few studies indicating which responding cellular components mediate granulomatous damage.

With respect to immune responses to other helminth phyla, the role of Th17 is less clear. An association between pathology and Th17 has been suggested in human filarial infection with patients exhibiting lymphedema caused by lymphatic filariasis having increased numbers of peripheral blood lymphocytes producing IL-17 along with decreased Tregs number (108). The presence of cytokines IL-1β, IL-23, and TGF-β have also been shown to augment these filarial-specific Th17 responses (109). Pulmonary hemorrhaging and neutrophilia caused by migration through the lung by *N. brasiliensis*, a rodent hookworm, was also shown to be dependent on IL-17 expression (110).

While the association of Th17 and IL-17 with pathology in helminth infection is robust, there is limited evidence of a role of Th17 in protection against helminths. One study, looking in blood cultures from patients who received praziquantel to clear *S. haematobium* infection demonstrated an association between high levels of Th17-associated cytokines (IL-21 and IL-23) with a decreased risk of re-infection (111). IL-17 expression has also been linked to both mucosal damage and hyper-contractility of the jejunum of *T. spiralis*-infected mice suggesting a role of Th17 in expulsion of the worms from the gut, but the study is highly preliminary and no depletion of IL-17 was attempted (112). *Echinostoma caproni* establishes a chronic infection in mice while rats are able to expel the worms after 4 weeks post-infection. Intestinal Th17-family cytokines IL-17, IL-23, and TGF-β were markedly upregulated in rats but not in mice, suggesting Th17 activation may be protective in this model (113). Conversely, an ovine model of *Teladorsagia circumcincta* infection demonstrated increased Th17 cytokines correlating with susceptibility to infection (114). Overall, the role of IL-17 producing Th17 cells in helminth driven immune responses is preliminary, and further work is needed.

## Th22 Cell in Helminth Infection

The cytokine IL-22 is normally associated with responses to microbes and its production mainly attributed to Th17 cells in both mice and human beings (115). However, a distinct subset of human skin CD4^+^ T-cells has recently been shown to produce IL-22 but not IL-17 or IFN-g (116–118), and thus has been given the term “Th22”. Th22 responses have been more widely studied in human beings than in mice so far, with a broad range of functional activities demonstrated, both proinflammatory and anti-inflammatory. While IL-22 is mainly produced by immune cells, the expression of its receptor IL-22R is mostly restricted to non-hematopoietic cells, such as epithelial cells (119). Th22 cells arise from the stimulation of naive T-cells in the presence of IL-6 and TNFα or presentation of antigen in the context of plasmacytoid dendritic cells, and appears to be independent of RORγt but dependent upon the aryl hydrocarbon receptor (AHR) (116, 120, 121).

To date, only a few studies have attempted to address the role of IL-22 in the context of helminth infection. IL-22 is upregulated in the intestinal mucosa after infection by *Trichuris trichuria* or *Necator americanus* in human beings (122–124) and Th22 frequency in PBMC is higher in filarial-infected patients than in healthy controls (109). While helminth infection clearly induces IL-22, so far no role for Th22 in either immune-mediated protection or pathology has been proven. In fact, IL-22^−/−^ mice infected with *S. mansoni* did not present significantly modified immune responses compared to wild type controls, neither did the absence of IL-22 modify the establishment of the parasite or the development of pathology (125). In filarial infection, Th22 frequency in PBMC was higher in lymphedema-positive people than in asymptomatic people, as was their frequency after antigen restimulation with both adult and microfilarial stages of the parasite (109). As reported in the above section, IL22 produced by Th17 cells plays a role in gut expulsion of *N. brasiliensis* and *T. muris* (126).

Th22 has also been reported to be involved in skin repair mechanisms and as such may be relevant to the pathology following skin penetration by helminths. Furthermore, IL-22 is known to have a role in the control of dysbiosis in the gut (127). As helminths have co-evolved with both the host and its microbiome (128), in order to observe the role of Th22 in helminth infection, it may be required to study the tripartite interaction of microbiome–macrobiome–host rather that the classical bipartite helminth–host interaction.

## Tfh Cell Subset in Helminth Infection

In distinction to the other CD4^+^ T-helper cell subsets, Tfh cells were not initially described based on cytokine production and transcription factor expression patterns, but rather by the expression of the surface marker CXCR5^+^ denoting its localization to the germinal center of human tonsils. Tfh have since been shown to promote germinal center formation and class switching of B-cells in mice and are further characterized by their expression of the inducible co-stimulatory molecule (ICOS), the inhibitory receptor programed cell death-1 (PD-1), and B and T lymphocyte attenuator (BLTA) (129). Bcl6 has been identified as the master transcription regulator for Tfh cells (130–132).

In the context of *N. brasiliensis* infection, Tfh cells have been demonstrated to express IL-4 but not IL-13 (20). This study also reports that these IL-4^+^ Tfh cells localize to the B-cells follicle, and not to tissue sites such as the lungs. Interestingly, work by Glatman Zaretsky et al. demonstrated that IL-4-expressing CXCR5^+^ Tfh cells could develop from adoptively transferred Th2 cells (CXCR5^-^ and PD1^-^) in B-cell-sufficient hosts during *S. mansoni* infection (133). Furthermore, IL-4 production by Tfh cells is essential for proper B-cell expansion and activation, as demonstrated by a reduction in B-cell activation in IL-4R^−/−^ mice (21).

The role of Tfh was studied in the context of *S. japonicum* induced pathology model (134). In ICOSL^−/−^ mice that are deficient in Tfh cells, a diminution of the liver pathology is associated with a decrease in Th1 and Tregs, whereas Th2 and Th17 are unaffected. Furthermore, adoptive transfer of Tfh cells to ICOSL^−/−^ mice proved sufficient to re-establish pathology characterized by the accumulation of cells in granulomas in the liver (134). Furthermore, plasma cells have been shown to be present in the granuloma induced by *S. japonicum* infection in both pig and mice, and depletion of B-cells reduced pathology in this model (135, 136).

In the context of non-helminth infections, Tfh cells have been shown to produce IFN-γ and several studies suggest that Tfh cell production of Th1-, Th2-, and Th17-associated cytokines provides further evidence that they are potentially derived from these lineages (129). Whether these different patterns of cytokine expression reflect different subsets of Tfh cells, akin to those observed in T-helper subsets and ILCs, is still unclear. The use of Bcl6 reporter mice may help to distinguish Tfh cells from other T-helper subsets and to provide an answer to this question, and may allow the identification of other cytokine patterns in Tfh cells that are induced in helminth infection in parallel with the other T-helper subsets.

## Hypothetical ThGM Cell Subset in Helminth Infection?

ThGM are the most recently proposed CD4^+^ T-helper subset having been described in *in vitro* studies developing from naïve CD4^+^ T-cells stimulated with anti-CD3 and anti-CD28, in the absence of IL-4, IFN-γ, and IL-12 (137). It is important to note that to our knowledge, this subset has not been described *in vivo* so far and that its existence will have to be further confirmed.

Putative ThGM cells produce high levels of GM-CSF, while not producing Th1- or Th2-associated cytokines. The authors further show that they do not express T-bet, GATA3, RorγT, or Foxp3, thus supporting the idea that they indeed constitute a new CD4^+^ T-helper subset (137). GM-CSF is a pluripotent cytokine, which has been shown to induce T-cells proliferation and activate macrophages and neutrophils, among other cells, and the absence of this cytokine has been shown to negatively impact the differentiation of both Th1 and Th2 responses (138).

Contrasting roles have been shown for GM-CSF in helminth infection settings. In *N. brasiliensis* infection, mice deficient in GM-CSF show no reduction in worm burden in the lungs or gut in both primary and secondary infection when compared to wild type mice (139).

In *Onchocerca volvulus* infection, presumably immune individuals (negative for the parasite, but living in endemic area) have a mixed Th1/Th2 response to L3 and microfilariae antigen, contrary to infected individuals that present only a strong Th2 response to those antigens. In particular, GM-CSF, at that time considered as a Th1 cytokine, was greatly enhanced in the putatively immune individuals (140). Co-cultivating human PBMCs *in vitro* with the *Schistosoma* antigen SmGST28 has been shown to be sufficient to induce some granulomatous formations, and GM-CSF is needed for this reaction (141), suggesting a role for this cytokine in development of pathology.

## Further T-Helper Cell Diversity and Plasticity in Helminth-Induced Type 2 Immune Responses

In the original Th1/Th2 paradigm, it was proposed that the T-helper cell subsets were distinct and negatively regulated each others’ activities, all underpinned by regulatory epigenetic methylation signatures interacting at IFN-γ, *IL4*, Gata-3, and T-bet gene loci (142, 143). However, this concept has to be revised in light of the recent discoveries of additional functionally diverse T-helper subsets including those with mixed Th1/Th2 signature cytokine phenotypes and by the observation that certain T-helper subsets can reverse their degree of polarization (144, 145).

Although it has been known for some time that double-positive IFN-γ^+^IL-4^+^ T-cells can be detected in experimental models of Th2 differentiation (146), when viewed in the context of the original Th1/Th2 paradigm, they were considered to be Th0 cells that were not yet committed to a polarized phenotype. However, several recent publications clearly demonstrated that these Th1/Th2 hybrid cells are stable both *in vitro* and *in vivo* after infection with either the trematode *S. mansoni* and the nematode *H. polygyrus* helminth infections (146, 147). Furthermore, this Th1/Th2 hybrid cells arise in a IL-18-dependent manner in *S. mansoni* infected mice (148). Interestingly, adoptive transfer of Th1/Th2 hybrid into Th1 or Th2 inflammatory models (LCMV and allergic airway inflammation) showed in both cases a reduced pathology associated with the inflammation (147). At the molecular level, these Th1/Th2 hybrid cells present with an intermediate expression of Gata-3 and T-bet as compared to Th2 and Th1 cells, respectively, due to an intermediate signature of methylation, for example, gata3 methylation was 36% in those cells, versus 60% in Th1 and 8% of Th2 (149).

**Figure 2:**
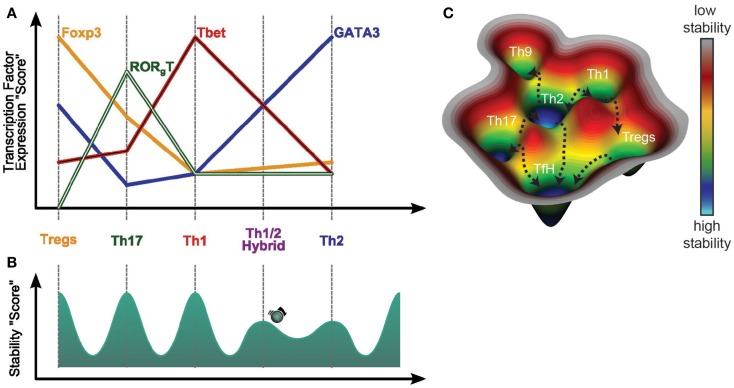
**Dynamic of T helper differentiation**. **(A)** The dynamic of T helper differentiation can be visualized as a “potential landscape” in which each T helper subset represent a stable position or “valley” and the transition from one subset to another, would be a “hill”, difficult to pass. Initially, the transition state between T helper subsets was considered as instable, and thus not observable *in vivo*. However, Th1/Th2 hybrid population has recently been reported to be stable after helminth infection. As this hybrid state is less abundant than Th1 or Th2, one could presume that the hybrid population is less stable that the Th1 or Th2 subsets, thus represented as a less deep well. **(B)** This transition between subsets can be further defined by the ration of transcription factor participating in the fate determination of each subset. For example, the Th1/Th2 population has been shown to present intermediary level of gata-3 and T-bet expression as compared respectively to Th2 and Th1. Through similar transcriptomic approach, generalised on all the T helper subsets, it would thus be possible to define a ration of transcription factors necessary to enable the switch from one subset to another. **(C)** The plasticity of the T helper subsets is represented in a conceptual 3D potential landscape and illustrate that the diverse repertoire of T helper cell subsets, and its important plasticity, enable the host to have an array of fine-tuned adaptive responses to both control the parasite development and avoid and repair pathology caused by the worm migration.

Both T-bet and GATA3 can regulate each others expression (150), and in a recent study, RORγt and Foxp3 were shown to directly interact in a way that determines Th17 versus induced Treg lineage (151, 152). Also, it has been shown that Tfh cells can express both Gata3 or T-bet and that while Bcl6 decreases their relative expression it does not block it completely (153). Taking these observations together, it would seem reasonable to speculate that it is the ratio of transcription factors induced that may determine the fate of any developing T-helper subset and that as a T-helper cell differentiates there is available a broad contiguous range of gene expression patterns for shaping its ultimate phenotype during an immune response against a helminth (Figure 2A) (154).

The possibility of potential inter-conversions of different T-helper subsets in the context of a helminth infection has recently been studied (155). Both *in vitro*- and *ex vivo*-generated Th1 and Th17 cells, adoptively transferred into mice later infected with *N. brasiliensis*, were shown to convert into IL-4 producers while losing their own signature cytokine expression. Also, both the iTregs and the nTregs were found to be stable *in vivo* in this study, with only low number of cells converting to express IL-4 (155). It is interesting to note that the *in vitro*-generated Th1 and Th17 cells showed a lower propensity to convert after transfer, suggesting that *in vitro* culture has a strong impact on the degree of T-helper cell plasticity that can be observed and perhaps explaining why the initial *in vitro* investigations into T-helper subset differentiation found subsets were highly stable and thus terminally differentiated.

Based on observations of the diversity and apparent plasticity of T-helper subsets phenotypes that can be detected now and on dynamic systems theory, we propose a landscape representation of the possibility of inter-conversions and intermediary states for T-helper cell subsets developing in the context of a helminth-induced immune response (Figures 2B,C). We have represented the dynamic of T-cells’ fates on a quasi-potential landscape in which the different subsets constitute stable states, also called attractors. In this view, the subsets are in “valley” or “flat” areas. To transit from one stable state to another, the system needs to be perturbed, for example by an infection that would push cells toward certain attractors or away from others. Unstable states are usually represented as “hills.” The Th1/Th2 hybrid state is stable enough, but less that the more terminally differentiated Th1 and Th2, and is thus represented by a well with less depth than Th1 or Th2.

Using such a “continuum of T-helper cell phenotypes” paradigm, it would be predicted that in host tissues responding to helminths there would be a gradient of activated and differentiated T-helper cell subsets with the most fully differentiated being stable and having lost much of their plasticity. Such terminally differentiated helper T-cells, maybe such as Th2, probably represent a small proportion of the pool of memory effector T-cells that maintain the helminth antigen specificity and the appropriate cytokine profile.

## Type 2 Immune Responses to Helminth Infection are a Compromise between Protection, Susceptibility, Tissue Repair, and Pathology

The original paradigm explaining resistance or susceptibility to helminths was described as a simple balance between Th1 (the susceptible, pathologic response), and Th2 (the response conferring parasite killing and elimination). However, this paradigm did not satisfactorily explain why so many parasites are able to establish themselves in hosts for extended periods of time (sometimes for decades) without causing any major clinical symptoms, nor why, in endemic environments, hosts are continually being reinfected with no apparent sign of disease. The hypothesis that the immune system would ignore the parasite infection was unconvincing, begging the question of whether these parasites themselves actively downregulate the host immune response and control pathology. Consequently, much research on helminth infection has focused on understanding how the parasite could regulate immune responses and teasing apart what is the physiological purpose of the type 2 effector responses in terms of benefit to host survival.

With the emergence of data showing that helminths and their products could be used to prevent/cure both allergic and autoimmune (156), our understanding of the immune response against helminth has changed to take into account the regulatory mechanisms induced by the parasites. From this emerged a new concept, the “modified Th2,” characterized by a decrease IL-5 and IL-13 expression and an increase of anti-inflammatory cytokines, such as TGF-b and IL-10. Other cells types, such as regulatory myeloid cells or regulatory B-cells have been shown to be involved in the downregulation induced by helminth.

The Th2 immune response has also been shown to be part of a wound repair response, with the ability to block a runaway pathology. This is clearly illustrated in IL-4^−/−^ mice infected with *S. mansoni* that die from excess pathology, even if their worm burden was similar to the wild type control mice (157). Th2 immune response has been considered as having evolved to cope both with helminth infection and with damage repair mechanisms, necessary to the survival of the host against those multicellular metazoan parasite migrating through tissue (12, 158). New research has shown that IL-9 has roles in controlling fibrosis and wound repair (91, 92), and that IL-22 from Th22 and Th17 cells promotes healing by increasing proliferation and survival of keratinocytes and intestinal epithelial cells (118, 159, 160). These data indicate that Th2 may not be the only T-helper subset contributing to the repair of helminth-induced tissue damage.

Furthermore, ES-62, a secretory product of the filarial nematode *Acanthocheilonema viteae*, may play with the balance of the different T-helper subsets in order to diminish the protective immune response (161). In a murine model of OVA-induced allergic airways inflammation, ES-62 decreased Th2 responses, as shown by a reduced level of IL-4 in the broncho-alveolar fluid, decrease eosinophils recruitment, and IgE. In parallel, the Th1 immune response is exacerbated, and neutralization of IFN-γ initiated the allergic inflammation blocked by ES-62 treatment. Th17 was also suppressed in this model, and that this suppression is responsible for the increase of the Th1 response. Importantly, ES-62 in this model did not induce an increase of Treg population, thus suggesting that the immunomodulatory effect of the molecule is to modify the balance of CD4^+^ T-helper subsets. This is the key data to understanding that the regulation of immune responses is not solely controlled by Tregs, but the different CD4^+^ T-cells subsets themselves antagonize one another.

**Figure 3:**
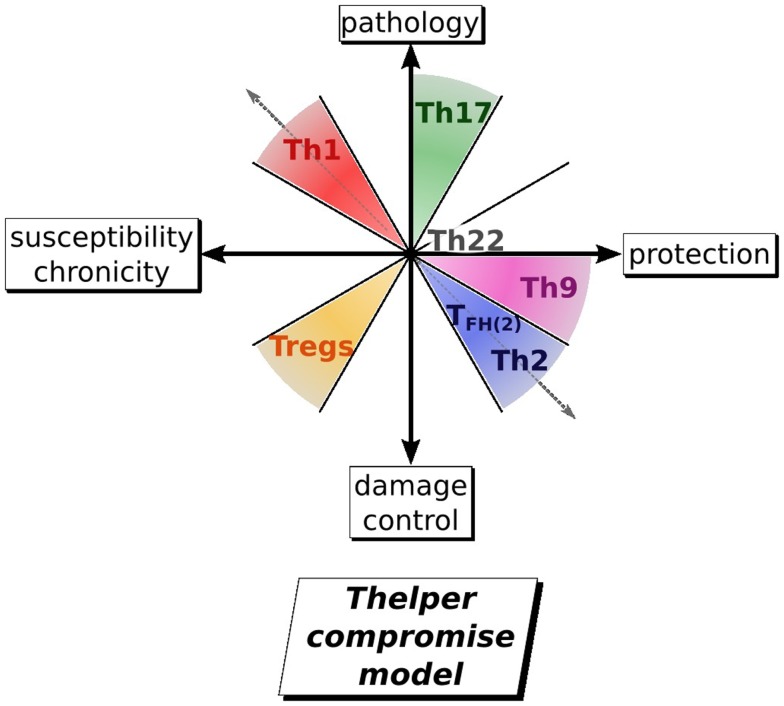
**Evolution of the view on T helper involvement in helminth infection**. By taking into account all the other T helper subset known to date, it is proposed that an immune response against on helminth can be summarized as a 2D map defined by an axis of susceptibility/protection and an axis of pathology/damage control. For an optimal response against a parasite, the host would thus mount a Th2/Th9 response with a low Tregs response and almost not existing Th1, Th17 response. The Th2 arm of the immune response protects against helminth by expanding ILCs, eosinophils and basophils all involved in parasite expulsion or by activating macrophages in AAM, playing a role in granuloma formation. Th9 rather protects by increasing goblet cells hyperplasia and muscle contractility in the gut. Th17 induced pathology is mainly mediated by neutrophils and inflammatory macrophages. In contrary, Tregs induced development of regulatory macrophages, which control pathology.

Considering the fact that all the currently recognized stable CD4^+^ T-helper phenotypes have been observed during helminth infections, and that these different subsets contribute variously to either protection, wound healing, susceptibility, or immunopathology, we have designed a new model that condenses what is known about the direct contributions of the subsets and their cross-regulation of each other. This model demonstrates our better understanding of the balanced immune responses during helminth infection Figure 3.

The early opposition of Th1 and Th2 is still clearly visible as a driving force for the trade-off between host and helminth survival. Th2 and Th9 segregate together to confer resistance, as they share a common activation pathway through IL-4. They are opposed to Th1 and Th17 in regard to pathology and to Th1 and Tregs in regard to susceptibility and chronicity. This model emphasizes that what may be best for the host is a compromise between elimination of the parasite versus containment and also the need for rapid repair of damaged tissue and avoidance of self destructive pathology. This all-encompassing view of an immune response gives a better understanding of the host issues at stake and gives a context for further investigations to investigate roles for each of the T-helper subsets. Furthermore, the consideration of a multipartite balance, rather than a one in one balance, would be useful to the design of therapy against helminths (i.e., by developing adjuvant to vaccine that could determine the right balance of Th susbsets to obtain sterilizing immunity, and define the timing to administer such treatment), and to understand how helminth infection or their product could use a therapy against inflammatory and autoimmune disease, caused by the deregulation of different T-helper subsets.

## Discussion/Remarks

The new challenge to the investigation of type 2 immune responses is to determine how many subsets of T-helper cells exist and what mechanisms control the level and degree of plasticity that occurs between T-helper cell subtypes. The question arises as to what would be the benefit to the host to have such complexity and myriad of genetic events underpinning this plasticity of the T-helper cell response. We would argue that the benefit to the host in being able to generate so many T-helper subsets is to have the diversity of options for dealing with the myriad of parasitic forms, invasive routes, and environments that have an endless supply of parasites that invade by physical means. In effect, the diversity and plasticity of the repertoire of functional T-helper cell subsets enable the host to have an array of adaptive responses. While the response might not kill the worm, it will enable the host to repair the more serious damage caused by the migrating parasite, and avoid the fatal consequences of debilitating pathology.

We wish to point out that in order to study the type 2 immune response elicited by helminths, i.e., define the role of the various old and new discrete CD4^+^ T-cell subsets, both techniques and approaches will have to evolve. For example, as plasticity between different T-helper subsets become increasingly evident, it maybe of interest to define a subset by both the cytokine production/non-production patterns, as well as by the ratio of transcription factors they express. For this matter, engineered reporter mice for particular cytokines and even combinations of cytokines may help in *in vivo* studies but may not reflect the native mRNA or protein. New bioinformatics approaches, such as studies of the transittability (162) (that defines the lower number of molecule switch to go from one fate of differentiation to another one based on a network of molecules involved in shaping the cell fate), could provide in the near future a list of more appropriate marker necessary to define one particular T-helper subset. Moreover, due to the plasticity of the T-cells, we think it is important to remember that immune studies look at dynamic events, and as such looking at a precise time point in the model, may give a wrong picture of the actual mechanism, for example a subset “in transition,” may be missed because of the lack of markers used for describing it, or the relative rarity compared with the currently defined stable subsets.

Hopefully, advances in single cell analysis (Fluidigm), sequencing, multiplex quantification of transcripts (such as nCounter, Nanostring that can detect up to 800 genes), advanced multicolor flow cytometry (such as panels up to 20 colors), the emergence of mass cytometry (such as CyTOF that allows multi-detection of up to 34 parameters to date, but could potentially go up to 100), coupled with bioinformatic approaches may offer the new tools necessary for studying the dynamics of T-helper differentiation in the context of helminth infection.

## Conflict of Interest Statement

The authors declare that the research was conducted in the absence of any commercial or financial relationships that could be construed as a potential conflict of interest.

## References

[1] Pond L Wassom DL Hayes CE Evidence for differential induction of helper T cell subsets during *Trichinella spiralis* infection. J Immunol (1989) 143:4232–7 2531779

[2] Kopf M Le Gros G Bachmann M Lamers MC Bluethmann H Köhler G Disruption of the murine IL-4 gene blocks Th2 cytokine responses. Nature (1993) 362:245–8 10.1038/362245a0 8384701

[3] Urban JF Katona IM Paul WE Finkelman FD Interleukin 4 is important in protective immunity to a gastrointestinal nematode infection in mice. Proc Natl Acad Sci U S A (1991) 88:5513–7 10.1073/pnas.88.13.5513 2062833 PMC51907

[4] Urban JF Noben-Trauth N Donaldson DD Madden KB Morris SC Collins M IL-13, IL-4Ralpha, and Stat6 are required for the expulsion of the gastrointestinal nematode parasite *Nippostrongylus brasiliensis*. Immunity (1998) 8:255–64 10.1016/S1074-7613(00)80477-X 9492006

[5] Harvie M Camberis M Tang S-C Delahunt B Paul W Le Gros G The lung is an important site for priming CD4 T-cell-mediated protective immunity against gastrointestinal helminth parasites. Infect Immun (2010) 78:3753–62 10.1128/IAI.00502-09 20605978 PMC2937440

[6] Else KJ Finkelman FD Maliszewski CR Grencis RK Cytokine-mediated regulation of chronic intestinal helminth infection. J Exp Med (1994) 179:347–51 10.1084/jem.179.1.347 8270879 PMC2191309

[7] Brunet LR Kopf MA Pearce EJ *Schistosoma mansoni*: IL-4 is necessary for concomitant immunity in mice. J Parasitol (1999) 85:734–6 10.2307/3285752 10461958

[8] Rawat J Dixon JB Macintyre AR McGarry HF Taylor MJ IL-4 dependent resistance to the tapeworm *Mesocestoides corti* (Cestoda) in mice. Parasite Immunol (2003) 25:553–7 10.1111/j.0141-9838.2004.00666.x 15053776

[9] Harris N Gause WC To B or not to B: B cells and the Th2-type immune response to helminths. Trends Immunol (2011) 32:80–8 10.1016/j.it.2010.11.005 21159556 PMC3076625

[10] Rodriguez-Sosa M David JR Bojalil R Satoskar AR Terrazas LI Cutting edge: susceptibility to the larval stage of the helminth parasite taenia crassiceps is mediated by Th2 response induced via STAT6 signaling. J Immunol (2002) 168:3135–9 10.4049/jimmunol.168.7.3135 11907063

[11] Wynn TA IL-13 effector functions. Annu Rev Immunol (2003) 21:425–56 10.1146/annurev.immunol.21.120601.141142 12615888

[12] Allen JE Sutherland TE Host protective roles of type 2 immunity: parasite killing and tissue repair, flip sides of the same coin. Semin Immunol (2014) 26:329–40 10.1016/j.smim.2014.06.003 25028340 PMC4179909

[13] Anthony RM Urban JF Alem F Hamed HA Rozo CT Boucher J-L Memory T(H)2 cells induce alternatively activated macrophages to mediate protection against nematode parasites. Nat Med (2006) 12:955–60 10.1038/nm1451 16892038 PMC1955764

[14] Rothenberg ME Hogan SP The eosinophil. Annu Rev Immunol (2006) 24:147–74 10.1146/annurev.immunol.24.021605.090720 16551246

[15] Behm CA Ovington KS The role of eosinophils in parasitic helminth infections: insights from genetically modified mice. Parasitol Today (2000) 16:202–9 10.1016/S0169-4758(99)01620-8 10782080

[16] Meeusen EN Balic A Do eosinophils have a role in the killing of helminth parasites? Parasitol Today (2000) 16:95–101 10.1016/S0169-4758(99)01607-5 10689327

[17] Martin C Al-Qaoud KM Ungeheuer MN Paehle K Vuong PN Bain O IL-5 is essential for vaccine-induced protection and for resolution of primary infection in murine filariasis. Med Microbiol Immunol (2000) 189:67–74 10.1007/PL00008258 11138639

[18] Kurowska-Stolarska M Kewin P Murphy G Russo RC Stolarski B Garcia CC IL-33 induces antigen-specific IL-5+ T cells and promotes allergic-induced airway inflammation independent of IL-4. J Immunol (2008) 181:4780–90 10.4049/jimmunol.181.7.4780 18802081

[19] Anuradha R George PJ Hanna LE Chandrasekaran V Kumaran PP Nutman TB Parasite-antigen driven expansion of IL-5(-) and IL-5(+) Th2 human subpopulations in lymphatic filariasis and their differential dependence on IL-10 and TGFβ. PLoS Negl Trop Dis (2014) 8:e2658 10.1371/journal.pntd.0002658 24498448 PMC3907332

[20] Liang H-E Reinhardt RL Bando JK Sullivan BM Ho I-C Locksley RM Divergent expression patterns of IL-4 and IL-13 define unique functions in allergic immunity. Nat Immunol (2012) 13:58–66 10.1038/ni.2182 22138715 PMC3242938

[21] King IL Mohrs M IL-4-producing CD4+ T cells in reactive lymph nodes during helminth infection are T follicular helper cells. J Exp Med (2009) 206:1001–7 10.1084/jem.20090313 19380638 PMC2715031

[22] Le Gros G Ben-Sasson SZ Seder R Finkelman FD Paul WE Generation of interleukin 4 (IL-4)-producing cells in vivo and in vitro: IL-2 and IL-4 are required for in vitro generation of IL-4-producing cells. J Exp Med (1990) 172:921–9 10.1084/jem.172.3.921 2117636 PMC2188542

[23] Brewer JM Conacher M Hunter CA Mohrs M Brombacher F Alexander J Aluminium hydroxide adjuvant initiates strong antigen-specific Th2 responses in the absence of IL-4- or IL-13-mediated signaling. J Immunol (1999) 163:6448–54 10586035

[24] Jankovic D Kullberg MC Noben-Trauth N Caspar P Paul WE Sher A Single cell analysis reveals that IL-4 receptor/Stat6 signaling is not required for the in vivo or in vitro development of CD4+ lymphocytes with a Th2 cytokine profile. J Immunol (2000) 164:3047–55 10.4049/jimmunol.164.6.3047 10706693

[25] van Panhuys N Tang S-C Prout M Camberis M Scarlett D Roberts J In vivo studies fail to reveal a role for IL-4 or STAT6 signaling in Th2 lymphocyte differentiation. Proc Natl Acad Sci U S A (2008) 105:12423–8 10.1073/pnas.0806372105 18719110 PMC2527927

[26] Allen JE Maizels RM Diversity and dialogue in immunity to helminths. Nat Rev Immunol (2011) 11:375–88 10.1038/nri2992 21610741

[27] Constant SL Bottomly K Induction of Th1 and Th2 CD4+ T cell responses: the alternative approaches. Annu Rev Immunol (1997) 15:297–322 10.1146/annurev.immunol.15.1.297 9143690

[28] Milner JD Fazilleau N McHeyzer-Williams M Paul W Cutting edge: lack of high affinity competition for peptide in polyclonal CD4+ responses unmasks IL-4 production. J Immunol (2010) 184:6569–73 10.4049/jimmunol.1000674 20495070 PMC2930602

[29] Shiner EK Holbrook BC Alexander-Miller MA CD4+ T cell subset differentiation and avidity setpoint are dictated by the interplay of cytokine and antigen mediated signals. PLoS One (2014) 9:e100175 10.1371/journal.pone.0100175.g006 24940899 PMC4062528

[30] Rudulier CD McKinstry KK Al-Yassin GA Kroeger DR Bretscher PA The number of responding CD4 T cells and the dose of antigen conjointly determine the Th1/Th2 phenotype by modulating B7/CD28 interactions. J Immunol (2014) 192:5140–50 10.4049/jimmunol.1301691 24752446

[31] van Panhuys N Klauschen F Germain RN T-cell-receptor-dependent signal intensity dominantly controls CD4. Immunity (2014) 41:63–74 10.1016/j.immuni.2014.06.003 24981853 PMC4114069

[32] Camberis M Prout M Tang S-C Forbes-Blom E Robinson M Kyle R Evaluating the in vivo Th2 priming potential among common allergens. J Immunol Methods (2013) 394:62–72 10.1016/j.jim.2013.05.004 23688767

[33] Breuilh L Vanhoutte F Fontaine J van Stijn CMW Tillie-Leblond I Capron M Galectin-3 modulates immune and inflammatory responses during helminthic infection: impact of galectin-3 deficiency on the functions of dendritic cells. Infect Immun (2007) 75:5148–57 10.1128/IAI.02006-06 17785480 PMC2168304

[34] Everts B Hussaarts L Driessen NN Meevissen MHJ Schramm G van der Ham AJ Schistosome-derived omega-1 drives Th2 polarization by suppressing protein synthesis following internalization by the mannose receptor. J Exp Med (2012) 209:1753–67–S1 10.1084/jem.20111381 22966004 PMC3457738

[35] Huber S Hoffmann R Muskens F Voehringer D Alternatively activated macrophages inhibit T-cell proliferation by Stat6-dependent expression of PD-L2. Blood (2010) 116:3311–20 10.1182/blood-2010-02-271981 20625006

[36] Jankovic D Kullberg MC Caspar P Sher A Parasite-induced Th2 polarization is associated with down-regulated dendritic cell responsiveness to Th1 stimuli and a transient delay in T lymphocyte cycling. J Immunol (2004) 173:2419–27 10.4049/jimmunol.173.4.2419 15294955

[37] Harris NL Peach RJ Ronchese F CTLA4-Ig inhibits optimal T helper 2 cell development but not protective immunity or memory response to *Nippostrongylus brasiliensis*. Eur J Immunol (1999) 29:311–6 10.1002/(SICI)1521-4141(199901)29:01<311::AID-IMMU311>3.0.CO;2-B 9933113

[38] Lu P Zhou X Chen SJ Moorman M Morris SC Finkelman FD CTLA-4 ligands are required to induce an in vivo interleukin 4 response to a gastrointestinal nematode parasite. J Exp Med (1994) 180:693–8 10.1084/jem.180.2.693 8046343 PMC2191583

[39] Balic A Harcus Y Holland MJ Maizels RM Selective maturation of dendritic cells by *Nippostrongylus brasiliensis*-secreted proteins drives Th2 immune responses. Eur J Immunol (2004) 34:3047–59 10.1002/eji.200425167 15468056

[40] MacDonald AS Straw AD Bauman B Pearce EJ CD8- dendritic cell activation status plays an integral role in influencing Th2 response development. J Immunol (2001) 167:1982–8 10.4049/jimmunol.167.4.1982 11489979

[41] Pletinckx K Stijlemans B Pavlovic V Laube R Brandl C Kneitz S Similar inflammatory DC maturation signatures induced by TNF or *Trypanosoma brucei* antigens instruct default Th2-cell responses. Eur J Immunol (2011) 41:3479–94 10.1002/eji.201141631 21928284

[42] Jankovic D Kullberg MC Hieny S Caspar P Collazo CM Sher A In the absence of IL-12, CD4(+) T cell responses to intracellular pathogens fail to default to a Th2 pattern and are host protective in an IL-10(-/-) setting. Immunity (2002) 16:429–39 10.1016/S1074-7613(02)00278-9 11911827

[43] Cervi L MacDonald AS Kane C Dzierszinski F Pearce EJ Cutting edge: dendritic cells copulsed with microbial and helminth antigens undergo modified maturation, segregate the antigens to distinct intracellular compartments, and concurrently induce microbe-specific Th1 and helminth-specific Th2 responses. J Immunol (2004) 172:2016–20 10.4049/jimmunol.172.4.2016 14764665

[44] Kane CM Cervi L Sun J McKee AS Masek KS Shapira S Helminth antigens modulate TLR-initiated dendritic cell activation. J Immunol (2004) 173:7454–61 10.4049/jimmunol.173.12.7454 15585871

[45] Everts B Perona-Wright G Smits HH Hokke CH van der Ham AJ Fitzsimmons CM Omega-1, a glycoprotein secreted by *Schistosoma mansoni* eggs, drives Th2 responses. J Exp Med (2009) 206:1673–80 10.1084/jem.20082460 19635864 PMC2722183

[46] Steinfelder S Andersen JF Cannons JL Feng CG Joshi M Dwyer D The major component in schistosome eggs responsible for conditioning dendritic cells for Th2 polarization is a T2 ribonuclease (omega-1). J Exp Med (2009) 206:1681–90 10.1084/jem.20082462 19635859 PMC2722182

[47] Whelan M Harnett MM Houston KM Patel V Harnett W Rigley KP A filarial nematode-secreted product signals dendritic cells to acquire a phenotype that drives development of Th2 cells. J Immunol (2000) 164:6453–60 10.4049/jimmunol.164.12.6453 10843701

[48] Connor L Tang S-C Camberis M Le Gros G Ronchese F Helminth–conditioned dendritic cells prime CD4+ T cells to IL-4 production in vivo. J Immunol (2014) 193:2709–17 10.4049/jimmunol.1400374 25108019

[49] Massacand JC Stettler RC Meier R Humphreys NE Grencis RK Marsland BJ Helminth products bypass the need for TSLP in Th2 immune responses by directly modulating dendritic cell function. Proc Natl Acad Sci U S A (2009) 106:13968–73 10.1073/pnas.0906367106 19666528 PMC2729004

[50] Hepworth MR Danilowicz-Luebert E Rausch S Metz M Klotz C Maurer M Mast cells orchestrate type 2 immunity to helminths through regulation of tissue-derived cytokines. Proc Natl Acad Sci U S A (2012) 109:6644–9 10.1073/pnas.1112268109 22493240 PMC3340035

[51] León B Ballesteros-Tato A Browning JL Dunn R Randall TD Lund FE Regulation of T(H)2 development by CXCR5+ dendritic cells and lymphotoxin-expressing B cells. Nat Immunol (2012) 13:681–90 10.1038/ni.2309 22634865 PMC3548431

[52] Tang H Cao W Kasturi SP Ravindran R Nakaya HI Kundu K The T helper type 2 response to cysteine proteases requires dendritic cell-basophil cooperation via ROS-mediated signaling. Nat Immunol (2010) 11:608–17 10.1038/ni.1883 20495560 PMC3145206

[53] Perrigoue JG Saenz SA Siracusa MC Allenspach EJ Taylor BC Giacomin PR MHC class II-dependent basophil-CD4+ T cell interactions promote T(H)2 cytokine-dependent immunity. Nat Immunol (2009) 10:697–705 10.1038/ni.1740 19465906 PMC2711559

[54] Yoshimoto T Yasuda K Tanaka H Nakahira M Imai Y Fujimori Y Basophils contribute to T(H)2-IgE responses in vivo via IL-4 production and presentation of peptide-MHC class II complexes to CD4+ T cells. Nat Immunol (2009) 10:706–12 10.1038/ni.1737 19465908

[55] Phythian-Adams AT Cook PC Lundie RJ Jones LH Smith KA Barr TA CD11c depletion severely disrupts Th2 induction and development in vivo. J Exp Med (2010) 207:2089–96 10.1084/jem.20100734 20819926 PMC2947067

[56] Epstein MM Di Rosa F Jankovic D Sher A Matzinger P Successful T cell priming in B cell-deficient mice. J Exp Med (1995) 182:915–22 10.1084/jem.182.4.915 7561694 PMC2192294

[57] Ronchese F Hausmann B Le Gros G Interferon-gamma- and interleukin-4-producing T cells can be primed on dendritic cells in vivo and do not require the presence of B cells. Eur J Immunol (1994) 24:1148–54 10.1002/eji.1830240521 8181524

[58] MacDonald AS Pearce EJ Cutting edge: polarized Th cell response induction by transferred antigen-pulsed dendritic cells is dependent on IL-4 or IL-12 production by recipient cells. J Immunol (2002) 168:3127–30 10.4049/jimmunol.168.7.3127 11907061

[59] Straw AD MacDonald AS Denkers EY Pearce EJ CD154 plays a central role in regulating dendritic cell activation during infections that induce Th1 or Th2 responses. J Immunol (2003) 170:727–34 10.4049/jimmunol.170.2.727 12517934

[60] Jenkins SJ Perona-Wright G Worsley AGF Ishii N MacDonald AS Dendritic cell expression of OX40 ligand acts as a costimulatory, not polarizing, signal for optimal Th2 priming and memory induction in vivo. J Immunol (2007) 179:3515–23 10.4049/jimmunol.179.6.3515 17785785

[61] Sun J Pearce EJ Suppression of early IL-4 production underlies the failure of CD4 T cells activated by TLR-stimulated dendritic cells to differentiate into Th2 cells. J Immunol (2007) 178:1635–44 10.4049/jimmunol.178.3.1635 17237413

[62] Flynn S Toellner KM Raykundalia C Goodall M Lane P CD4 T cell cytokine differentiation: the B cell activation molecule, OX40 ligand, instructs CD4 T cells to express interleukin 4 and upregulates expression of the chemokine receptor, Blr-1. J Exp Med (1998) 188:297–304 10.1084/jem.188.2.297 9670042 PMC2212448

[63] Ohshima Y Yang LP Uchiyama T Tanaka Y Baum P Sergerie M OX40 costimulation enhances interleukin-4 (IL-4) expression at priming and promotes the differentiation of naive human CD4(+) T cells into high IL-4-producing effectors. Blood (1998) 92:3338–45 9787171

[64] Ito T Wang Y-H Duramad O Hori T Delespesse GJ Watanabe N TSLP-activated dendritic cells induce an inflammatory T helper type 2 cell response through OX40 ligand. J Exp Med (2005) 202:1213–23 10.1084/jem.20051135 16275760 PMC2213234

[65] Chu DK Llop-Guevara A Walker TD Flader K Goncharova S Boudreau JE IL-33, but not thymic stromal lymphopoietin or IL-25, is central to mite and peanut allergic sensitization. J Allergy Clin Immunol (2013) 131:.e1–8 10.1016/j.jaci.2012.08.002 23006545

[66] Schmitz J Owyang A Oldham E Song Y Murphy E McClanahan TK IL-33, an interleukin-1-like cytokine that signals via the IL-1 receptor-related protein ST2 and induces T helper type 2-associated cytokines. Immunity (2005) 23:479–90 10.1016/j.immuni.2005.09.015 16286016

[67] de Jong EC Vieira PL Kalinski P Schuitemaker JHN Tanaka Y Wierenga EA Microbial compounds selectively induce Th1 cell-promoting or Th2 cell-promoting dendritic cells in vitro with diverse th cell-polarizing signals. J Immunol (2002) 168:1704–9 10.4049/jimmunol.168.4.1704 11823500

[68] Hung L-Y Lewkowich IP Dawson LA Downey J Yang Y Smith DE IL-33 drives biphasic IL-13 production for noncanonical type 2 immunity against hookworms. Proc Natl Acad Sci U S A (2013) 110:282–7 10.1073/pnas.1206587110 23248269 PMC3538196

[69] Mearns H Forbes-Blom EE Camberis M Tang S-C Kyle R Harvie M IL-25 exhibits disparate roles during Th2-cell differentiation versus effector function. Eur J Immunol (2014) 44:1976–80 10.1002/eji.201344400 24737448

[70] Pippig SD Peña-Rossi C Long J Godfrey WR Fowell DJ Reiner SL Robust B cell immunity but impaired T cell proliferation in the absence of CD134 (OX40). J Immunol (1999) 163:6520–9 10586044

[71] Ochiai S Roediger B Abtin A Shklovskaya E Fazekas de St Groth B Yamane H CD326loCD103loCD11blo dermal dendritic cells are activated by thymic stromal lymphopoietin during contact sensitization in mice. J Immunol (2014) 193:2504–11 10.4049/jimmunol.1400536 25057004

[72] Moser M Murphy KM Dendritic cell regulation of TH1-TH2 development. Nat Publish Group (2000) 1:199–205 10.1038/79734 10973276

[73] Denda-Nagai K Aida S Saba K Suzuki K Moriyama S Oo-puthinan S Distribution and function of macrophage galactose-type C-type lectin 2 (MGL2/CD301b): efficient uptake and presentation of glycosylated antigens by dendritic cells. J Biol Chem (2010) 285:19193–204 10.1074/jbc.M110.113613 20304916 PMC2885198

[74] Gao Y Nish SA Jiang R Hou L Licona-Limón P Weinstein JS Control of T helper 2 responses by transcription factor IRF4-dependent dendritic cells. Immunity (2013) 39:722–32 10.1016/j.immuni.2013.08.028 24076050 PMC4110745

[75] Kumamoto Y Linehan M Weinstein JS Laidlaw BJ Craft JE Iwasaki A CD301b^+^ dermal dendritic cells drive T helper 2 cell-mediated immunity. Immunity (2013) 39:733–43 10.1016/j.immuni.2013.08.029 24076051 PMC3819035

[76] Murakami R Denda-Nagai K Hashimoto S-I Nagai S Hattori M Irimura T A unique dermal dendritic cell subset that skews the immune response toward Th2. PLoS One (2013) 8:e73270 10.1371/journal.pone.0073270 24039898 PMC3767795

[77] Williams JW Tjota MY Clay BS Vander Lugt B Bandukwala HS Hrusch CL Transcription factor IRF4 drives dendritic cells to promote Th2 differentiation. Nat Commun (2013) 4:2990 10.1038/ncomms3990 24356538 PMC4003872

[78] Brown EM Arrieta M-C Finlay BB A fresh look at the hygiene hypothesis: how intestinal microbial exposure drives immune effector responses in atopic disease. Semin Immunol (2013) 25:378–87 10.1016/j.smim.2013.09.003 24209708

[79] Maizels R Balic A Gomez-Escobar N Nair M Taylor MD Allen JE Helminth parasites – masters of regulation. Immunol Rev (2004) 201:90–116 10.1111/j.0105-2896.2004.00191.x 15361235

[80] McSorley HJ Maizels RM Helminth infections and host immune regulation. Clin Microbiol Rev (2012) 25:585–608 10.1128/CMR.05040-11 23034321 PMC3485755

[81] Gessner A Blum H Röllinghoff M Differential regulation of IL-9-expression after infection with *Leishmania major* in susceptible and resistant mice. Immunobiology (1993) 189:419–35 10.1016/S0171-2985(11)80414-6 8125519

[82] Faulkner H Humphreys N Renauld JC Van Snick J Grencis R Interleukin-9 is involved in host protective immunity to intestinal nematode infection. Eur J Immunol (1997) 27:2536–40 10.1002/eji.1830271011 9368607

[83] Faulkner H Renauld JC Van Snick J Grencis RK Interleukin-9 enhances resistance to the intestinal nematode *Trichuris muris*. Infect Immun (1998) 66:3832–40 9673269 10.1128/iai.66.8.3832-3840.1998 PMC108429

[84] Veldhoen M Uyttenhove C van Snick J Helmby H Westendorf A Buer J Transforming growth factor-beta “reprograms” the differentiation of T helper 2 cells and promotes an interleukin 9-producing subset. Nat Immunol (2008) 9:1341–6 10.1038/ni.1659 18931678

[85] Staudt V Bothur E Klein M Lingnau K Reuter S Grebe N Interferon-regulatory factor 4 is essential for the developmental program of T helper 9 cells. Immunity (2010) 33:192–202 10.1016/j.immuni.2010.07.014 20674401

[86] Uyttenhove C Brombacher F van Snick J TGF-β interactions with IL-1 family members trigger IL-4-independent IL-9 production by mouse CD4(+) T cells. Eur J Immunol (2010) 40:2230–5 10.1002/eji.200940281 20540113

[87] Richard M Grencis RK Humphreys NE Renauld JC Van Snick J Anti-IL-9 vaccination prevents worm expulsion and blood eosinophilia in *Trichuris muris*-infected mice. Proc Natl Acad Sci U S A (2000) 97:767–72 10.1073/pnas.97.2.767 10639154 PMC15405

[88] Khan WI Richard M Akiho H Blennerhasset PA Humphreys NE Grencis RK Modulation of intestinal muscle contraction by interleukin-9 (IL-9) or IL-9 neutralization: correlation with worm expulsion in murine nematode infections. Infect Immun (2003) 71:2430–8 10.1128/IAI.71.5.2430-2438.2003 12704113 PMC153239

[89] Licona-Limón P Henao-Mejia J Temann AU Gagliani N Licona-Limón I Ishigame H Th9 cells drive host immunity against gastrointestinal worm infection. Immunity (2013) 39:744–57 10.1016/j.immuni.2013.07.020 24138883 PMC3881610

[90] Turner J-E Morrison PJ Wilhelm C Wilson M Ahlfors H Renauld J-C IL-9-mediated survival of type 2 innate lymphoid cells promotes damage control in helminth-induced lung inflammation. J Exp Med (2013) 210:2951–65 10.1084/jem.20130071 24249111 PMC3865473

[91] Re Lo S Lison D Huaux F CD4+ T lymphocytes in lung fibrosis: diverse subsets, diverse functions. J Leukoc Biol (2013) 93:499–510 10.1189/jlb.0512261 23159927

[92] Gerlach K Hwang Y Nikolaev A Atreya R Dornhoff H Steiner S TH9 cells that express the transcription factor PU.1 drive T cell-mediated colitis via IL-9 receptor signaling in intestinal epithelial cells. Nat Immunol (2014) 15:676–86 10.1038/ni.2920 24908389

[93] Harrington LE Hatton RD Mangan PR Turner H Murphy TL Murphy KM Interleukin 17-producing CD4+ effector T cells develop via a lineage distinct from the T helper type 1 and 2 lineages. Nat Publish Group (2005) 6:1123–32 10.1038/ni1254 16200070

[94] Basso AS Cheroutre H Mucida D More stories on Th17 cells. Cell Res (2009) 19:399–411 10.1038/cr.2009.26 19255592 PMC2838708

[95] Sie C Korn T Mitsdoerffer M Th17 cells in central nervous system autoimmunity. Exp Neurol (2014). 10.1016/j.expneurol.2014.03.009 24681001

[96] Wüthrich M Deepe GS Klein B Adaptive immunity to fungi. Annu Rev Immunol (2012) 30:115–48 10.1146/annurev-immunol-020711-074958 22224780 PMC3584681

[97] Mathew RC Boros DL Anti-L3T4 antibody treatment suppresses hepatic granuloma formation and abrogates antigen-induced interleukin-2 production in *Schistosoma mansoni* infection. Infect Immun (1986) 54:820–6 3096893 10.1128/iai.54.3.820-826.1986 PMC260243

[98] Phillips SM DiConza JJ Gold JA Reid WA Schistosomiasis in the congenitally athymic (nude) mouse. I. Thymic dependency of eosinophilia, granuloma formation, and host morbidity. J Immunol (1977) 118:594–9 839071

[99] Stadecker MJ Asahi H Finger E Hernandez HJ Rutitzky LI Sun J The immunobiology of Th1 polarization in high-pathology schistosomiasis. Immunol Rev (2004) 201:168–79 10.1111/j.0105-2896.2004.00197.x 15361240

[100] Rutitzky LI Lopes da Rosa JR Stadecker MJ Severe CD4 T cell-mediated immunopathology in murine schistosomiasis is dependent on IL-12p40 and correlates with high levels of IL-17. J Immunol (2005) 175:3920–6 10.4049/jimmunol.175.6.3920 16148138

[101] Shainheit MG Smith PM Bazzone LE Wang AC Rutitzky LI Stadecker MJ Dendritic cell IL-23 and IL-1 production in response to schistosome eggs induces Th17 cells in a mouse strain prone to severe immunopathology. J Immunol (2008) 181:8559–67 10.4049/jimmunol.181.12.8559 19050275 PMC2663362

[102] Shainheit MG Lasocki KW Finger E Larkin BM Smith PM Sharpe AH The pathogenic Th17 cell response to major schistosome egg antigen is sequentially dependent on IL-23 and IL-1β. J Immunol (2011) 187:5328–35 10.4049/jimmunol.1101445 22003203 PMC3653625

[103] Tallima H Salah M Guirguis FR Ridi El R Transforming growth factor-beta and Th17 responses in resistance to primary murine schistosomiasis mansoni. Cytokine (2009) 48:239–45 10.1016/j.cyto.2009.07.581 19717308

[104] Zhang Y Chen L Gao W Hou X Gu Y Gui L IL-17 neutralization significantly ameliorates hepatic granulomatous inflammation and liver damage in *Schistosoma japonicum* infected mice. Eur J Immunol (2012) 42:1523–35 10.1002/eji.201141933 22678906

[105] Chen D Luo X Xie H Gao Z Fang H Huang J Characteristics of IL-17 induction by *Schistosoma japonicum* infection in C57BL/6 mouse liver. Immunology (2013) 139:523–32 10.1111/imm.12105 23551262 PMC3719069

[106] Chen D Xie H Luo X Yu X Fu X Gu H Roles of Th17 cells in pulmonary granulomas induced by *Schistosoma japonicum* in C57BL/6 mice. Cell Immunol (2013) 285:149–57 10.1016/j.cellimm.2013.09.008 24212062

[107] Mbow M Larkin BM Meurs L Wammes LJ de Jong SE Labuda LA T-helper 17 cells are associated with pathology in human schistosomiasis. J Infect Dis (2013) 207:186–95 10.1093/infdis/jis654 23087431 PMC3571236

[108] Babu S Bhat SQ Pavan Kumar N Lipira AB Kumar S Karthik C Filarial lymphedema is characterized by antigen-specific Th1 and th17 proinflammatory responses and a lack of regulatory T cells. PLoS Negl Trop Dis (2009) 3:e420 10.1371/journal.pntd.0000420 19381284 PMC2666805

[109] Anuradha R George PJ Chandrasekaran V Kumaran PP Nutman TB Babu S Interleukin 1 (IL-1)- and IL-23-mediated expansion of filarial antigen-specific Th17 and Th22 cells in filarial lymphedema. Clin Vaccine Immunol (2014) 21:960–5 10.1128/CVI.00257-14 24807054 PMC4097448

[110] Chen F Liu Z Wu W Rozo C Bowdridge S Millman A An essential role for TH2-type responses in limiting acute tissue damage during experimental helminth infection. Nat Med (2012) 18:260–6 10.1038/nm.2628 22245779 PMC3274634

[111] Bourke CD Nausch N Rujeni N Appleby LJ Mitchell KM Midzi N Integrated analysis of innate, Th1, Th2, Th17, and regulatory cytokines identifies changes in immune polarisation following treatment of human schistosomiasis. J Infect Dis (2013) 208:159–69 10.1093/infdis/jis524 23045617 PMC3666130

[112] Fu Y Wang W Tong J Pan Q Long Y Qian W Th17: a new participant in gut dysfunction in mice infected with *Trichinella spiralis*. Mediators Inflamm (2009) 2009:517052 10.1155/2009/517052 20016839 PMC2786920

[113] Sotillo J Trelis M Cortes A Fried B Marcilla A Esteban JG Th17 responses in *Echinostoma caproni* infections in hosts of high and low compatibility. Exp Parasitol (2011) 129:307–11 10.1016/j.exppara.2011.08.004 21875583

[114] Gossner AG Venturina VM Shaw DJ Pemberton JM Hopkins J Relationship between susceptibility of blackface sheep to *Teladorsagia circumcincta* infection and an inflammatory mucosal T cell response. Vet Res (2012) 43:26 10.1186/1297-9716-43-26 22455366 PMC3422184

[115] Acosta-Rodriguez EV Rivino L Geginat J Jarrossay D Gattorno M Lanzavecchia A Surface phenotype and antigenic specificity of human interleukin 17-producing T helper memory cells. Nat Publish Group (2007) 8:639–46 10.1038/ni1467 17486092

[116] Duhen T Geiger R Jarrossay D Lanzavecchia A Sallusto F Production of interleukin 22 but not interleukin 17 by a subset of human skin-homing memory T cells. Nat Immunol (2009) 10:857–63 10.1038/ni.1767 19578369

[117] Trifari S Kaplan CD Tran EH Crellin NK Spits H Identification of a human helper T cell population that has abundant production of interleukin 22 and is distinct from T(H)-17, T(H)1 and T(H)2 cells. Nat Immunol (2009) 10:864–71 10.1038/ni.1770 19578368

[118] Eyerich S Eyerich K Pennino D Carbone T Nasorri F Pallotta S Th22 cells represent a distinct human T cell subset involved in epidermal immunity and remodeling. J Clin Invest (2009) 119:3573–85 10.1172/JCI40202 19920355 PMC2786807

[119] Akdis M Palomares O Van De Veen W van Splunter M Akdis CA TH17 and TH22 cells: a confusion of antimicrobial response with tissue inflammation versus protection. J Allergy Clin Immunol (2012) 129:quiz1450–1 10.1016/j.jaci.2012.05.003 22657405

[120] Ramirez J-M Brembilla NC Sorg O Chicheportiche R Matthes T Dayer J-M Activation of the aryl hydrocarbon receptor reveals distinct requirements for IL-22 and IL-17 production by human T helper cells. Eur J Immunol (2010) 40:2450–9 10.1002/eji.201040461 20706985

[121] Veldhoen M Hirota K Christensen J O’Garra A Stockinger B Natural agonists for aryl hydrocarbon receptor in culture medium are essential for optimal differentiation of Th17 T cells. J Exp Med (2009) 206:43–9 10.1084/jem.20081438 19114668 PMC2626686

[122] Broadhurst MJ Leung JM Kashyap V McCune JM Mahadevan U McKerrow JH IL-22+ CD4+ T cells are associated with therapeutic trichuris trichiura infection in an ulcerative colitis patient. Sci Transl Med (2010) 2:60ra88 10.1126/scitranslmed.3001500 21123809

[123] Mcsorley HJ Gaze S Daveson J Jones D Anderson RP Clouston A Suppression of inflammatory immune responses in celiac disease by experimental hookworm infection. PLoS One (2011) 6:e24092 10.1371/journal.pone.0024092 21949691 PMC3174943

[124] Gaze S Mcsorley HJ Daveson J Jones D Bethony JM Oliveira LM Characterising the mucosal and systemic immune responses to experimental human hookworm infection. PLoS Pathog (2012) 8:e1002520 10.1371/journal.ppat.1002520.s003 22346753 PMC3276555

[125] Wilson MS Feng CG Barber DL Yarovinsky F Cheever AW Sher A Redundant and pathogenic roles for IL-22 in mycobacterial, protozoan, and helminth infections. J Immunol (2010) 184:4378–90 10.4049/jimmunol.0903416 20220096 PMC3170015

[126] Turner J-E Stockinger B Helmby H IL-22 mediates goblet cell hyperplasia and worm expulsion in intestinal helminth infection. PLoS Pathog (2013) 9:e1003698 10.1371/journal.ppat.1003698.g006 24130494 PMC3795034

[127] Leung JM Loke P A role for IL-22 in the relationship between intestinal helminths, gut microbiota and mucosal immunity. Int J Parasitol (2013) 43:253–7 10.1016/j.ijpara.2012.10.015 23178750 PMC3955947

[128] Parker W Ollerton J Evolutionary biology and anthropology suggest biome reconstitution as a necessary approach toward dealing with immune disorders. Evol Med Public Health (2013) 2013:89–103 10.1093/emph/eot008 24481190 PMC3868394

[129] King C New insights into the differentiation and function of T follicular helper cells. Nat Rev Immunol (2009) 9:757–66 10.1038/nri2644 19855402

[130] Johnston RJ Poholek AC DiToro D Yusuf I Eto D Barnett B Bcl6 and Blimp-1 are reciprocal and antagonistic regulators of T follicular helper cell differentiation. Science (2009) 325:1006–10 10.1126/science.1175870 19608860 PMC2766560

[131] Nurieva RI Chung Y Martinez GJ Yang XO Tanaka S Matskevitch TD Bcl6 mediates the development of T follicular helper cells. Science (2009) 325:1001–5 10.1126/science.1176676 19628815 PMC2857334

[132] Yu D Rao S Tsai LM Lee SK He Y Sutcliffe EL The transcriptional repressor Bcl-6 directs T follicular helper cell lineage commitment. Immunity (2009) 31:457–68 10.1016/j.immuni.2009.07.002 19631565

[133] Glatman Zaretsky A Taylor JJ King IL Marshall FA Mohrs M Pearce EJ T follicular helper cells differentiate from Th2 cells in response to helminth antigens. J Exp Med (2009) 206:991–9 10.1084/jem.20090303 19380637 PMC2715032

[134] Chen X Yang X Li Y Zhu J Zhou S Xu Z Follicular helper T cells promote liver pathology in mice during *Schistosoma japonicum* infection. PLoS Pathog (2014) 10:e1004097 10.1371/journal.ppat.1004097.s008 24788758 PMC4006917

[135] Hurst MH Willingham AL Lindberg R Experimental schistosomiasis japonica in the pig: immunohistology of the hepatic egg granuloma. Parasite Immunol (2002) 24:151–9 10.1046/j.1365-3024.2002.00448.x 11982860

[136] Ji F Liu Z Cao J Li N Liu Z Zuo J B cell response is required for granuloma formation in the early infection of *Schistosoma japonicum*. PLoS One (2008) 3:e1724 10.1371/journal.pone.0001724 18320044 PMC2248706

[137] Zhang J Roberts AI Liu C Ren G Xu G Zhang L A novel subset of helper T cells promotes immune responses by secreting GM-CSF. Cell Death Differ (2013) 20:1731–41 10.1038/cdd.2013.130 24076588 PMC3824596

[138] Jean WC Spellman SR Wallenfriedman MA Flores CT Kurtz BP Hall WA Effects of combined granulocyte-macrophage colony-stimulating factor (GM-CSF), interleukin-2, and interleukin-12 based immunotherapy against intracranial glioma in the rat. J Neurooncol (2004) 66:39–49 10.1023/B:NEON.0000013477.94568.0f 15015768

[139] Shim DSC Schilter HC Knott ML Almeida RA Short RP Mackay CR Protection against *Nippostrongylus brasiliensis* infection in mice is independent of GM-CSF. Immunol Cell Biol (2012) 90:553–8 10.1038/icb.2011.69 21844882

[140] Brattig N Nietz C Hounkpatin S Lucius R Seeber F Pichlmeier U Differences in cytokine responses to onchocerca volvulus extract and recombinant Ov33 and OvL3-1 proteins in exposed subjects with various parasitologic and clinical states. J Infect Dis (1997) 176:838–42 10.1086/517317 9291349

[141] Rezende CMF Goes TS Goes VS Azevedo V Leite MF Goes AM GM-CSF and TNF-alpha synergize to increase in vitro granuloma size of PBMC from humans induced by *Schistosoma mansoni* recombinant 28-kDa GST. Immunol Lett (2004) 95:221–8 10.1016/j.imlet.2004.07.015 15388264

[142] Allan RS Zueva E Cammas F Schreiber HA Masson V Belz GT An epigenetic silencing pathway controlling T helper 2 cell lineage commitment. Cell Mol Immunol (2012) 487:249–53 10.1038/nature11173 22763435

[143] Robinson M McConnell MJ Le Gros G How epigenetic imprinting contributes to stabilizing the Th2 phenotype. Immunol Cell Biol (2012) 90:917–8 10.1038/icb.2012.47 23010874

[144] Hirahara K Poholek A Vahedi G Laurence A Kanno Y Milner JD Mechanisms underlying helper T-cell plasticity: implications for immune-mediated disease. J Allergy Clin Immunol (2013) 131:1276–87 10.1016/j.jaci.2013.03.015 23622118 PMC3677748

[145] O’Shea JJ Paul WE Mechanisms underlying lineage commitment and plasticity of helper CD4+ T cells. Science (2010) 327:1098–102 10.1126/science.1178334 20185720 PMC2997673

[146] Miner KT Croft M Generation, persistence, and modulation of Th0 effector cells: role of autocrine IL-4 and IFN-gamma. J Immunol (1998) 160:5280–7 9605125

[147] Peine M Rausch S Helmstetter C Fröhlich A Hegazy AN Kühl AA Stable T-bet(+)GATA-3(+) Th1/Th2 hybrid cells arise in vivo, can develop directly from naive precursors, and limit immunopathologic inflammation. PLoS Biol (2013) 11:e1001633 10.1371/journal.pbio.1001633 23976880 PMC3747991

[148] Adachi K Nakamura R Osada Y Senba M Tamada K Hamano S Involvement of IL-18 in the expansion of unique hepatic T cells with unconventional cytokine profiles during *Schistosoma mansoni* infection. PLoS One (2014) 9:e96042 10.1371/journal.pone.0096042 24824897 PMC4019514

[149] Deaton AM Cook PC De Sousa D Phythian-Adams AT Bird A MacDonald AS A unique DNA methylation signature defines a population of IFN-γ/IL-4 double-positive T cells during helminth infection. Eur J Immunol (2014) 44:1835–41 10.1002/eji.201344098 24578067 PMC4231227

[150] Kanhere A Hertweck A Bhatia U Gökmen MR Perucha E Jackson I T-bet and GATA3 orchestrate Th1 and Th2 differentiation through lineage-specific targeting of distal regulatory elements. Nat Commun (2012) 3:1268 10.1038/ncomms2260 23232398 PMC3535338

[151] Zhou L Lopes JE Chong MMW Ivanov II Min R Victora GD TGF-beta-induced Foxp3 inhibits T(H)17 cell differentiation by antagonizing RORgammat function. Cell Mol Immunol (2008) 453:236–40 10.1038/nature06878 18368049 PMC2597437

[152] Ichiyama K Yoshida H Wakabayashi Y Chinen T Saeki K Nakaya M Foxp3 inhibits RORgammat-mediated IL-17A mRNA transcription through direct interaction with RORgammat. J Biol Chem (2008) 283:17003–8 10.1074/jbc.M801286200 18434325

[153] Lu KT Kanno Y Cannons JL Handon R Bible P Elkahloun AG Functional and epigenetic studies reveal multistep differentiation and plasticity of in vitro-generated and in vivo-derived follicular T helper cells. Immunity (2011) 35:622–32 10.1016/j.immuni.2011.07.015 22018472 PMC3235706

[154] O’Garra A Gabryšová L Spits H Quantitative events determine the differentiation and function of helper T cells. Nat Immunol (2011) 12:288–94 10.1038/ni.2003 21423225

[155] Panzer M Sitte S Wirth S Drexler I Sparwasser T Voehringer D Rapid in vivo conversion of effector T cells into Th2 cells during helminth infection. J Immunol (2012) 188:615–23 10.4049/jimmunol.1101164 22156341

[156] Maizels RM McSorley HJ Smyth DJ Helminths in the hygiene hypothesis: sooner or later? Clin Exp Immunol (2014) 177:38–46 10.1111/cei.12353 24749722 PMC4089153

[157] Brunet LR Finkelman FD Cheever AW Kopf MA Pearce EJ IL-4 protects against TNF-alpha-mediated cachexia and death during acute schistosomiasis. J Immunol (1997) 159:777–85 9218595

[158] Gause WC Wynn TA Allen JE Type 2 immunity and wound healing: evolutionary refinement of adaptive immunity by helminths. Nat Rev Immunol (2013) 13:607–14 10.1038/nri3476 23827958 PMC3789590

[159] Pickert G Neufert C Leppkes M Zheng Y Wittkopf N Warntjen M STAT3 links IL-22 signaling in intestinal epithelial cells to mucosal wound healing. J Exp Med (2009) 206:1465–72 10.1084/jem.20082683 19564350 PMC2715097

[160] Sonnenberg GF Fouser LA Artis D Border patrol: regulation of immunity, inflammation and tissue homeostasis at barrier surfaces by IL-22. Nat Immunol (2011) 12:383–90 10.1038/ni.2025 21502992

[161] Rzepecka J Siebeke I Coltherd JC Kean DE Steiger CN Al-Riyami L The helminth product, ES-62, protects against airway inflammation by resetting the Th cell phenotype. Int J Parasitol (2013) 43:211–23 10.1016/j.ijpara.2012.12.001 23291461 PMC3584281

[162] Wu F-X Wu L Wang J Liu J Chen L Transittability of complex networks and its applications to regulatory biomolecular networks. Sci Rep (2014) 4:4819 10.1038/srep04819 24769565 PMC4001102

